# Interfacial Role of Dopant *d*‐States on Hot Carrier Generation in Bimetallic Nanoparticles

**DOI:** 10.1002/nap2.70184

**Published:** 2026-07-01

**Authors:** Rania Zaier, Katarzyna Kluczyk‐Korch, Tomasz J. Antosiewicz

**Affiliations:** ^1^ Faculty of Physics University of Warsaw Warsaw Poland; ^2^ Department of Physics and Astronomy Chalmers University of Technology Gothenburg Sweden

## Abstract

Metallic nanoparticles are of great interest due to a collective localized surface plasmon resonance (LSPR), which decays through Landau damping and results in a nonthermal distribution of electrons and holes, so‐called hot carriers, the generation and harnessing of which are significant for photocatalysis, photovoltaics, and sensing. Bimetallic nanoparticles give the opportunity to tune their electronic structure to selectively produce hot carriers with desired energies. Using time‐dependent density functional theory, we study magnesium nanoparticles doped with Ag, Au, Cu, and Pd to investigate how the different electronic structure of dopant atoms impacts the LSPR and plasmonic hot carrier generation. Although the photoabsorption of the nanoparticle is only slightly affected by dopant atoms, the hot carrier energy distribution significantly depends on the dopant types and their spatial distribution. Specifically, interface dopants enhance hot carrier generation more than those located deeper within the dopant domain, highlighting the role of spatial arrangement in heterostructured nanoparticles. These findings demonstrate that bimetallic nanoparticles exhibit promising performance depending on the constituent elements and their spatial distribution, which opens up the possibility of tuning the generation of hot carriers with appropriate‐sized dopant clusters and interfaces through the generation and decay of LSPRs.

## Graphical abstract

Plasmonic heterostructures enhance hot carrier generation and spatial separation by deliberate doping with selected transition metal atoms. By deterministically structuring the spatial distribution of a set number of dopants, their d‐states can be engineered to result in tailored hot carrier energy distributions in selected spatial locations while using the localized surface plasmon resonance for efficient light absorption.

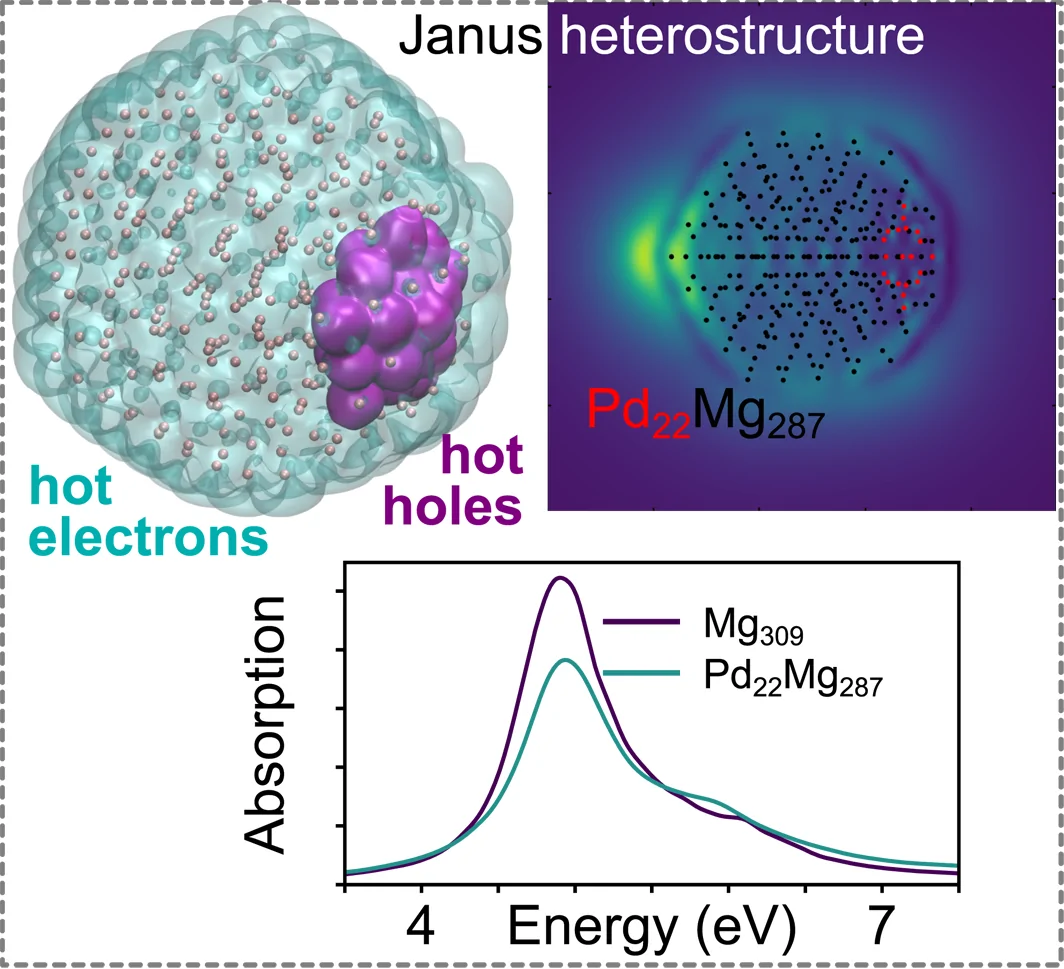

## 1 Introduction

Many recent advanced technologies in light harvesting and solar energy conversion rely on the unique properties of plasmonic nanoparticles (NPs) to generate hot carriers [1, 2]. Efficient light absorption by plasmonic NPs is enabled by a collective electronic excitation known as localized surface plasmon resonance (LSPR), which is particularly pronounced in noble metal NPs such as Ag or Au, in simple metals such as Al, Na, or Mg [3], or even in extended pi‐conjugated molecules [4]. When such NPs interact with molecules or semiconductor materials, they can modify the relevant parameters in ways that intensify light–matter interactions and thus accelerate various processes. In the field of photovoltaics, these hot carriers boost the electrical current production by improving the efficiency of sunlight‐to‐electricity conversion [5, 6]. The use of plasmonic NPs extends to other areas, such as photocatalysis by accelerating chemical reactions [7, 8, 9] or sensitive light detection across different wavelengths in photodetectors [10, 11].

The diversity of metallic NP applications highlights their significant impact on modern science and technology, driving innovation in a wide range of fields. For these applications, hot carriers follow two complementary pathways: hot holes drive oxidative reactions such as water oxidation, H_2_O_2_ production, and photodegradation of organic pollutants, whereas hot electrons promote reduction processes including H_2_ evolution, CO_2_ conversion to solar fuels, and charge injection into semiconductor supports [12, 13, 14, 15]. Beyond photocatalysis, hot electron transfer across Schottky barriers also contributes to photocurrent generation in photovoltaic and photodetection devices [16]. In bimetallic nanoparticles, the host drives plasmonic excitation and hot carrier generation, whereas the dopant governs catalytic activity and selectivity [17], a complementarity absent in single‐component nanoparticles.

LSPR decay, among other channels, occurs through Landau damping, a nonradiative process that involves transfer of energy from the plasmonic excitation to newly created electron‐hole pairs in the metal, resulting in the generation of hot carriers with significant energy. This process is influenced by the electronic structure of the material, in particular, by the hybridization of the Kohn–Sham (KS) states leading to tunable LSPRs [18], and the relative position of the sp and d bands of the metal. In particular, it was shown that the energy threshold for the d to sp transition can be tuned by changing the stoichiometric ratios of the doped Au/Cu [19] and Au/Ag [20] NPs. A density functional theory (DFT) study of various bimetallic NPs of noble and simple metals points to the impact of the dopants' electronic structure type: d‐closed shells (Ag, Au, Cu), d‐open (Pd), or s‐shaped (Na) on the hot electron energy distribution [21]. Similarly, numerical analysis of Au‐Ag NPs shows the impact of changes in heterostructure composition on the energy distribution of hot carriers, encouraging the generation of highly energetic holes by targeting interband d‐ to sp‐state transitions and energetic electrons by increasing intraband sp‐ to sp‐state transitions [22].

The spatial arrangement of the material domains also plays an important role in the dynamics of the carriers in bimetallic NPs. It affects the rate of hot carrier generation [23, 24], the spatial distribution of carriers [25, 26], and their overall lifetime [27]. In particular, numerical calculations reveal a key impact of the layer arrangement in the bimetallic core–shell NPs on the hot hole/electron separation [25, 26]. Furthermore, a single substitution of a surface atom with a transition metal in noble metallic NPs was predicted to cause noticeable changes in overall electronic density of states (DOS) and hot carrier (HC) distributions [28]. This prediction is supported by an experimental study showing a higher enhancement of propylene formation rates using Cu‐Pt NP catalysts with a single Pt atom dopant than with dopants consisting of small Pt clusters [29]. On the other hand, experimental studies of Au‐Pd NPs point to the importance of a spatial overlap between the area of the highly enhanced electromagnetic field and the Pd domain on the catalytic performance of NPs [30]. Tailoring of catalytic activity by doping extends, naturally, to non‐noble bimetallic NPs, such as Pt‐Ni NPs employed for seawater hydrogen production [31, 32].

One notable developing strategy to tailor the spatial and energetic distribution of generated hot carriers in NPs is to combine two or more different metals, such as Ag, Au, Cu, or Pd, into one doped structure [33]. These elements are recognized not only for their plasmonic behavior but also for their optically achievable d electronic states. Moreover, their d bands differ in position and width, which allows for the systematic study and tuning of the d‐ to sp‐state transition rates and the resulting hot carrier energies [24]. Such doped or mixed‐composition NPs can be of various types. One approach utilizes larger NPs decorated with small clusters, and this is one of the typical scenarios in plasmonic catalysis where a larger optical antenna interacts with smaller satellites [34, 35, 36]. An alternative is to decorate plasmonic particles with single‐atom dopant sites [28, 29]. Herein, we follow the former approach.

In this study, we examine the impact of the d states introduced by different concentrations of dopant atoms and their spatial arrangement on the generation of hot carriers in doped nanostructures using a time‐dependent density functional theory (TDDFT) approach. For simplicity, we start with an upcoming plasmonic simple metal that does not have d‐states, that is, magnesium [37], in the form of an icosahedral Mg_309_ NP. This particle is subsequently doped with four different metals, having d‐states (i.e., Ag, Au, Cu, Pd), at various concentrations by replacing a number of Mg atoms, namely 11, 22, 33, or 44 with dopants in the form of the compact, few‐tens‐of‐dopant‐atoms clusters (Janus structures) and randomly distributed dopant atoms (random heterostructures). Direct comparison of Janus and random structures allows us to investigate the influence of the spatial localization of d‐states in NPs particularly at the host‐dopant interface, on hot‐carrier generation. Furthermore, using this systematic methodology, we perform a comprehensive analysis of the optical properties and hot carrier generation capabilities of these doped structures to elucidate the roles of dopant atoms and their spatial arrangement.

## 2 Methods

Briefly, we model light–matter interactions by employing the real‐time TDDFT (RT‐TDDFT) approach [38] based on the linear combination of atomic orbitals (LCAO) mode [39] as implemented in the open‐source GPAW package [40, 41]. The structures are relaxed using the Perdew–Burke–Ernzerhof (PBE) exchange‐correlation functional [42] and a grid spacing of 0.2 Å. The initial Mg_309_ NP was assembled and structurally relaxed until all atomic forces were smaller than 0.01 eV/Å. Next, the doped nanoparticles were constructed by replacing selected Mg atoms with the new one and then the NPs were further relaxed under the same conditions.

Ground states for TDDFT are calculated using the Gritsenko–van Leeuwen–van Lenthe–Baerends (GLLB‐sc) exchange correlation potential. This approach significantly improves the representation of d‐electron states in noble metals compared to the standard PBE method [43, 44]. For all atoms, the default p‐valence basis sets included in GPAW are used, as they contain the diffuse 3p functions, which are important for describing plasmon resonances [45]. The employed basis set for Mg explicitly includes only the two electrons in the 3s orbital, whereas the lower electrons are treated as a frozen core within PAW; conversely, for other atoms, the explicitly treated electrons are: for Pd 10 and for Ag, Au, and Cu 11. For real‐time TDDFT a grid spacing parameter of 0.3 Å is chosen to represent densities and potentials, and the molecules/particles are surrounded by a vacuum region of at least 8 Å. For the time propagation, we use a time step Δt of 15 attoseconds and a total propagation time of at least T=30 fs. The time‐dependent response is determined through adiabatic PBE computations. Photoabsorption spectra are obtained using the δ‐kick technique [46] under the linear‐response regime employing the dipole approximation to model light–matter interaction. The TDDFT code is combined with analysis tools [47] suited for the evaluation and visualization in the form of transition contribution map (TCM), of electron‐hole transition contributions to photoabsorption [47], and the calculation of hot carrier energy distributions [48]. VMD is used to visualize the spatial distributions of hot charge carriers [49].

## 3 Results and Discussion

The base plasmonic nanostructure used in this work is an icosahedral Mg NP composed of 309 atoms as shown in Figure 1a. When excited by a plane electromagnetic field, it responds by generating a localized plasmon resonance, whose induced electric field is aligned along the direction of polarization and yields a strongly enhanced field at two opposite corners. This is illustrated in Figure 1b. Next, we substitute several Mg atoms at one of those corners with dopant atoms selected for their d‐electron configurations, namely Ag, Au, Cu, and Pd. In this way, we aim to elucidate how the presence of d‐electron states affects the plasmonic properties of these hybrid NPs. We analyze a few variations of the number of dopant atoms (11, 22, 33, and 44 atoms), as shown in Figure 1c for the particular case of the Ag dopant. Here, we present relaxed structures for these four configurations for the so‐called Janus heterostructures where the two distinct atoms form two distinct domains. This type is specifically in contrast to random heterostructures where the dopant atoms are randomly distributed throughout the nanoparticle, which are considered here as a distinct alternative (random heterostructures, see Supporting Information (SI)).

**FIGURE 1:**
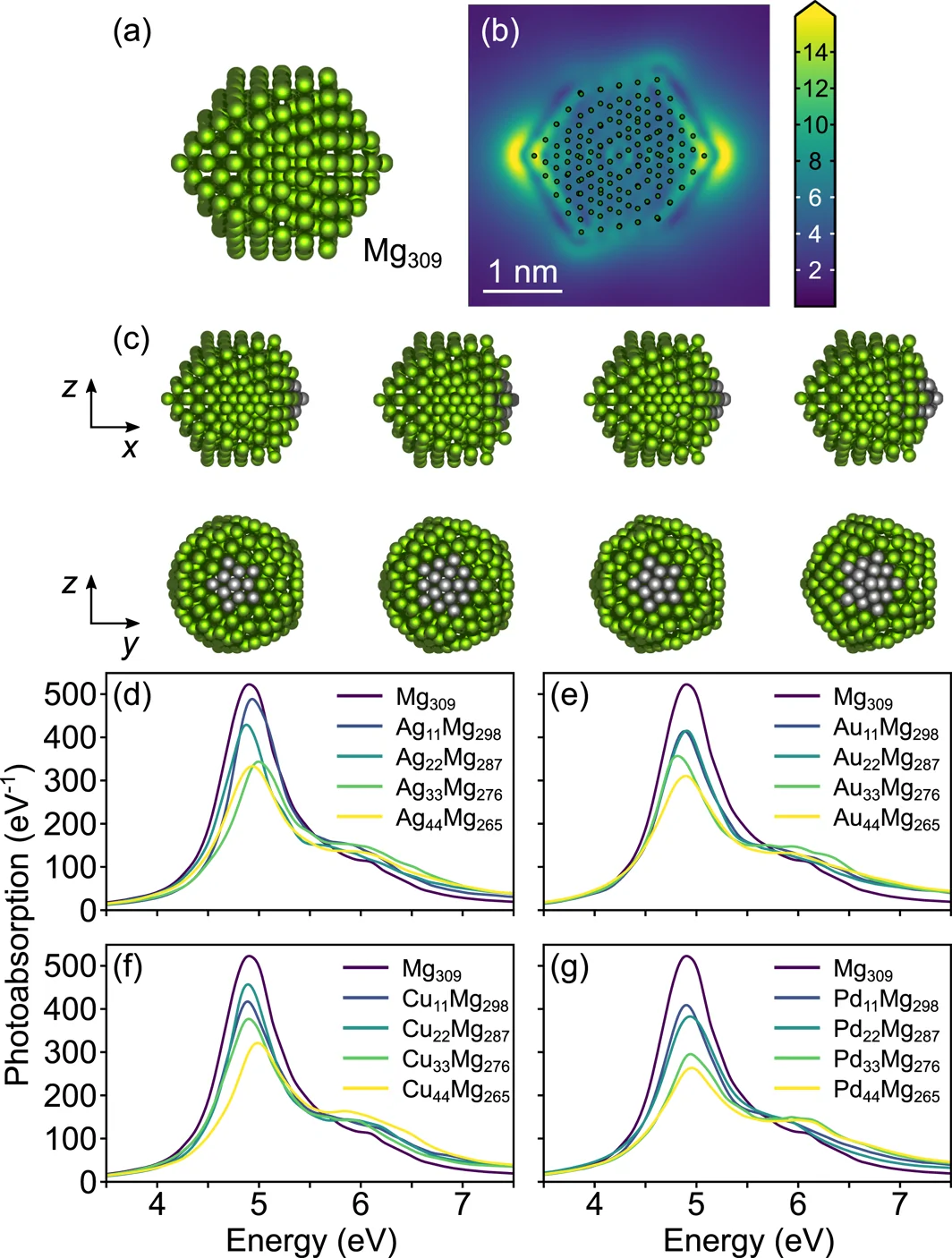
(a) Geometry of Mg_309_ NP and (b) the corresponding electromagnetic field enhancement showing an intense maxima at the two opposite corners along the x‐axis induced by an electric field with $E_{x}$ polarization. (c) Schematic illustration of Mg NP structures with single‐cluster Ag (silver) inclusions with increasing size in the xz and yz planes. The inclusions are placed at one of the NP ends that show large field enhancement for $E_{x}$ polarization. (d–g) Total photoabsorption spectra of the Mg_309_ plasmon and $A_{x}{\text{Mg}}_{309\mathrm{-x}}$ complexes for (d) Ag, (e) Au, (f) Cu, and (g) Pd dopant atoms. A significant reduction in plasmon intensity, accompanied by further spectral broadening, is observed after the introduction of dopant atoms.

The photoabsorption spectrum of magnesium NP exhibits a LSPR located at 4.90 eV (see Figure 1d–g) and a secondary shoulder around 6.00 eV. In both cases, these spectral features are caused by the collective oscillation of the valence electrons in Mg. The addition of dopant atoms to Mg_309_ NP causes a modification of the photoabsorption spectra. We observe a notable decrease of the intensity of the main peak and a slight increase and broadening of the intensity of the secondary peak. This apparent increase of the absorption in the secondary peak links it to the presence of the d to sp interband transitions associated with dopants [50]. Despite the fact that the total number of electrons involved into plasmon excitation increases with the increase of dopant concentration (as the dopant electrons contribute to LSPR), we observe a decrease of the main absorption peak. We relate this to the changes in the total DOS of the NP caused by the presence of d‐electron states in the dopants and the following introduction of additional plasmon decay paths [51]. We also observe a slight spectral shift of the LSPR energy both to the red and blue within the envelope of the Mg NP plasmon. These small variations are likely the result of minor differences in the NP geometry and conduction charge carrier density between NPs doped with different elements.

### 3.1 Transitions in Doped Nanoparticles

The introduction of dopant atoms leads to additional d‐states in the electronic structure of the Mg‐dopant NP. When incorporated into the host material, these dopant atoms interact with surrounding Mg atoms and disturb the electronic structure by forming new hybridized electronic states with changed energy levels [52, 53, 54, 55]. Additionally, within the doped structure, new mixed electronic transitions (host‐dopant or dopant–host) become possible. Therefore, we analyze the electronic transitions in terms of mixed and distinct (between states in the same atom type) ones depending on the initial and final‐states material weights, and how they contribute to photoabsorption. The material weight of an electronic state meets the following condition $w_{\text{Mg}}+w_{x}=1$ where $w_{\text{Mg}}$ is a sum over the individual contributions of Mg orbitals, and $w_{x}$ is a sum over the contributions of dopant atomic orbitals to this state. Subsequently, each transition is then decomposed into the individual sums of the initial and final states.

In Figure 2, we plot the fractional contributions to photoabsorption between the two distinct atom types: from Mg→Mg in the top plots of each subpanel and in the bottom plots of each subpanel Mg→dopant, dopant→Mg, and dopant→dopant, where the data are sorted by the number of dopant atoms, with the datasets offset by 15%. The smallest contribution comes from the dopant→dopant transitions, and for all considered cases, this fraction is typically less than 2% except for Pd doping, where it reaches almost 4%. However, when compared to the respective dopant fractions (e.g., 11 atoms of 309 is ca. 3.6%), it is clear that these dopant‐only transitions are under‐represented. Of the two types of mixed transitions (Mg→dopant or the reverse), both have, on average in the 4–7 eV range, comparable contributions. However, the Mg→dopant is relatively constant over the considered spectrum of interest, does not vary significantly between the different dopant atoms, and scales approximately linearly with the amount of dopant atoms (ca. 0.2%–0.4% per dopant atom). This constant behavior is most likely the result of the relative ratio of free carrier states in Mg versus in the dopants, which form the plasmon and are efficiently excited by light. The reverse transitions, namely dopant→Mg, show qualitatively different behavior in that they markedly increase in amplitude with energy. Their largest contribution is observed at the blue end of the spectrum for every dopant with Ag showing the smallest contribution followed by Cu, Au, and finally Pd exhibiting the largest transition to Mg. This prominent increase of dopant→Mg transitions at higher energies confirms the significant contribution of d‐states to observed increase of photoabsorption in this range. This onset, of course, depends strongly on the dopant. The distinct transitions of the host material (Mg‐Mg), which we single out in a separate panel due to a much larger magnitude, provide the dominant contributions to photoabsorption in the energy range of interest. At the minimum, these distinct transitions provide at least 60% of the oscillator strength to absorption above the Mg LSPR, whereas at lower energies, this fraction is even larger. The reduction of their contribution to photoabsorption at large energies is offset by the dopant→Mg transition.

**FIGURE 2:**
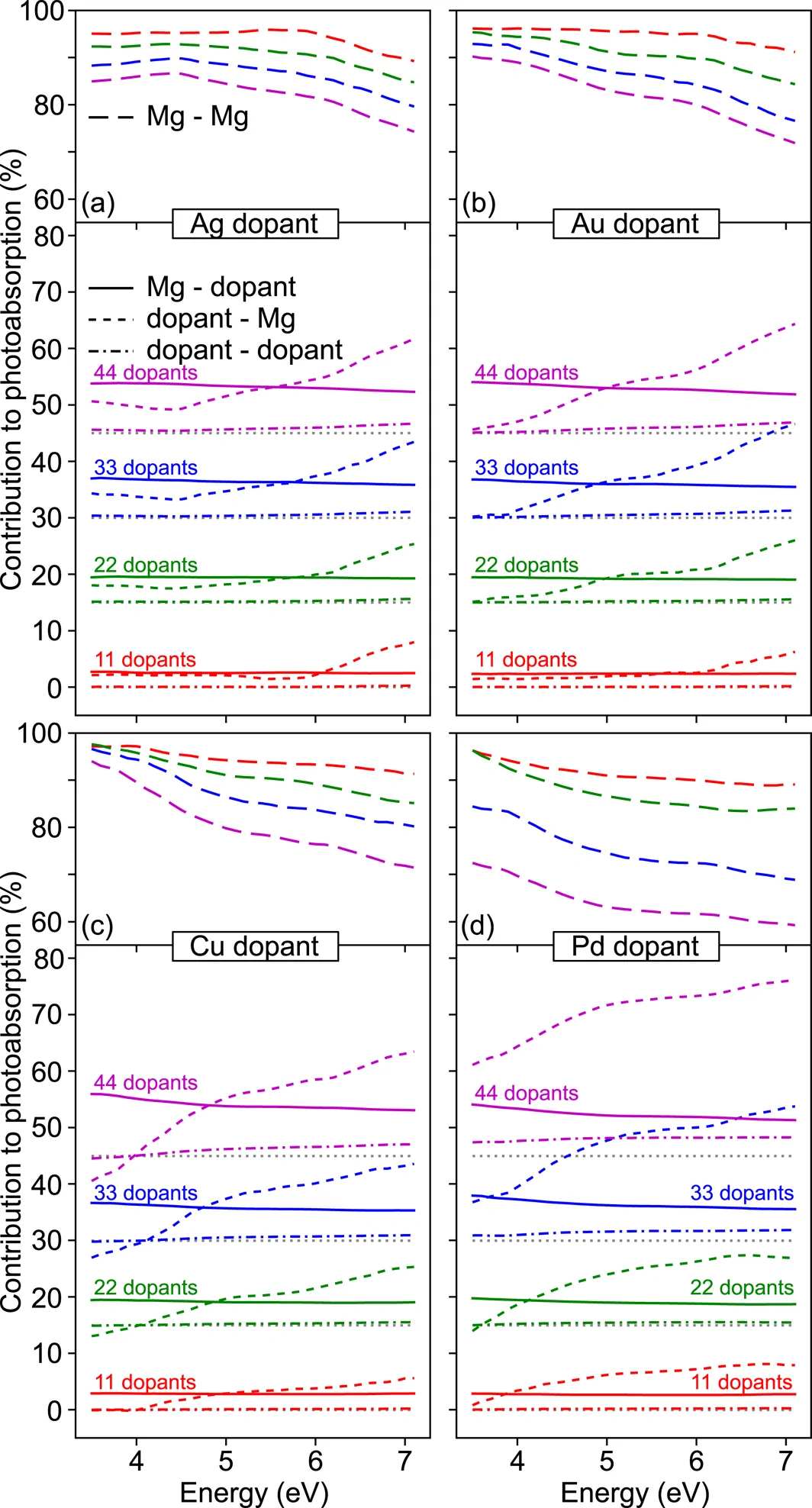
Contribution to photoabsorption of doped Janus NP heterostructures expressed as fraction of total absorption for transitions involving dopant atoms for (a) Ag dopant, (b) Au dopant, (c) Cu dopant, and (d) Pd dopant. In the top panel of each subplot—long dashes: Mg→Mg. In the bottom panel of each subplot—solid line: Mg→ dopant; dashed: dopant→Mg; dash‐dotted: dopant→dopant; colors indicate a particular number of dopant atoms. Each subsequent set is offset by 15% and its individual 0 is marked by a gray dotted line.

Next, we evaluate the efficiency of modifying photoabsorption spectra of the Mg‐add atom clusters by the dopant atoms. In Figure 3, we plot the photoabsorption spectra separated into only dopant atoms (dotted lines) and the mixed dopant→Mg transition, which are normalized to the number of dopant atoms. The spectra show the distinct plasmon around 5 eV and the large‐energy shoulder determined by the host NP material, which set the main absorption properties. The photoabsorption efficiency is the largest for the smallest amount of dopant in every case, both for dopant absorption as well as dopant→Mg transition and is in the range of 1 ${\text{eV}}^{-1}$. The efficiency gradually decreases as the doping level increases by up to a factor of 50% for a 4‐fold increase in the doping level. Hence, despite the efficiency decrease, increased doping up to the studied level has a positive net impact on both photoabsorption in the dopant atoms (as a possibility of generating hot electrons in the dopants) as well as the mixed dopant→Mg transition (to generate hot holes in the dopants). Interestingly, we note a significantly larger contribution to mixed transitions in the host Mg from Pd than the other materials. The variation in the contributions from mixed transitions can be assigned to the distinct electronic configurations and electronegativity of dopants originating from its electronic orbitals organization [52, 53, 54, 55]. Namely, the d‐electrons of these dopants contribute differently to electronic interactions within the Janus heterostructure than the replaced atoms, changing energy levels and modifying hybridization between host and dopant electronic states.

**FIGURE 3:**
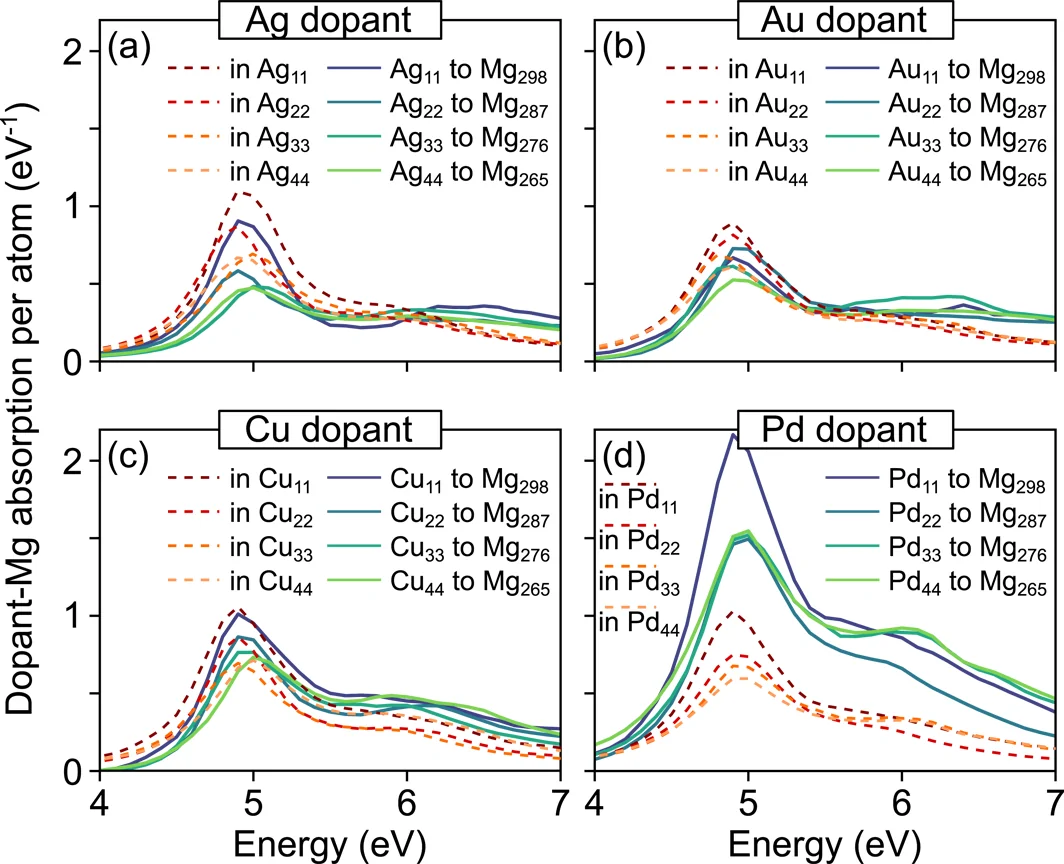
Efficiency of modifying photoabsorption spectra by doping presented as photoabsorption normalized to number of dopant atoms in Janus heterostructures. Dotted line: absorption →dopant (normalized total absorption in dopant); solid lines: dopant→Mg (only mixed transition). Generally, the best contribution per dopant atom is observed for the smallest number of dopants; however, the efficiency decrease is relatively small. It is at most a factor of 50% when going from 11 to 44 atoms, a 4‐fold increase.

In order to gain insight into the electronic properties of doped NPs, we calculate projected density of states (PDOS) with respect to d‐ and sp‐states. Calculations of the sp‐ and d‐orbitals were established based on the quantum number of the angular momentum $l_{\mu }$, which corresponds to the LCAO basis functions identified by the index μ [51]. For example, the sp‐ and d‐orbital contributions to the $n^{\text{th}}$ state are obtained as

(1) $$sp:\sum_{\mu :\;\;l_{\mu }=0,1}|C_{\mu n}^{\left(0\right)}{|}^{2},\;\;\;d:\sum_{\mu :\;\;l_{\mu }=2}|C_{\mu n}^{\left(0\right)}{|}^{2},$$

where the coefficients are normalized such that $\sum_{\mu }|C_{\mu n}^{\left(0\right)}{|}^{2}=1$. Here, our aim is to specifically focus on the influence of the d‐band electrons of the dopant atoms and their spatial arrangement, as understanding these changes in the PDOS as a function of the type, amount, and localization of dopants is crucial for designing and optimizing the electronic properties of NPs.

In Figure 4, we plot the PDOS of d‐ and sp‐states of the whole Janus heterostructure NPs with different dopants types and varying number of atoms. The mean d‐band energy gradually shifts from low to higher energies, as we change the dopant from Ag, through Au, and Cu to Pd. With increasing number of dopants atoms, the d‐state PDOS shows a gradual change from mostly distinct peaks into one broad band, whose intensity also increases with the number of dopants atoms. Furthermore, this broadening is governed by dopant spatial distribution, as the PDOS for random heterostructures, with corresponding number of dopant atoms, (see Supporting Information S1: Figure S2) shows a deep, narrow atom—like d‐states. The lack of development of a broader d‐band in the random heterostructures determines the possible energies of hot holes, which may not even be generated for low energy excitation. In contrast, clusters of dopant atoms allow for the formation of broader d bands in the structure with better mixing to the states of the host. The energy range and width of the bands depend on the dopant cluster size and by selecting the excitation energy one may decide on the energy of holes excited in the dopant clusters.

**FIGURE 4:**
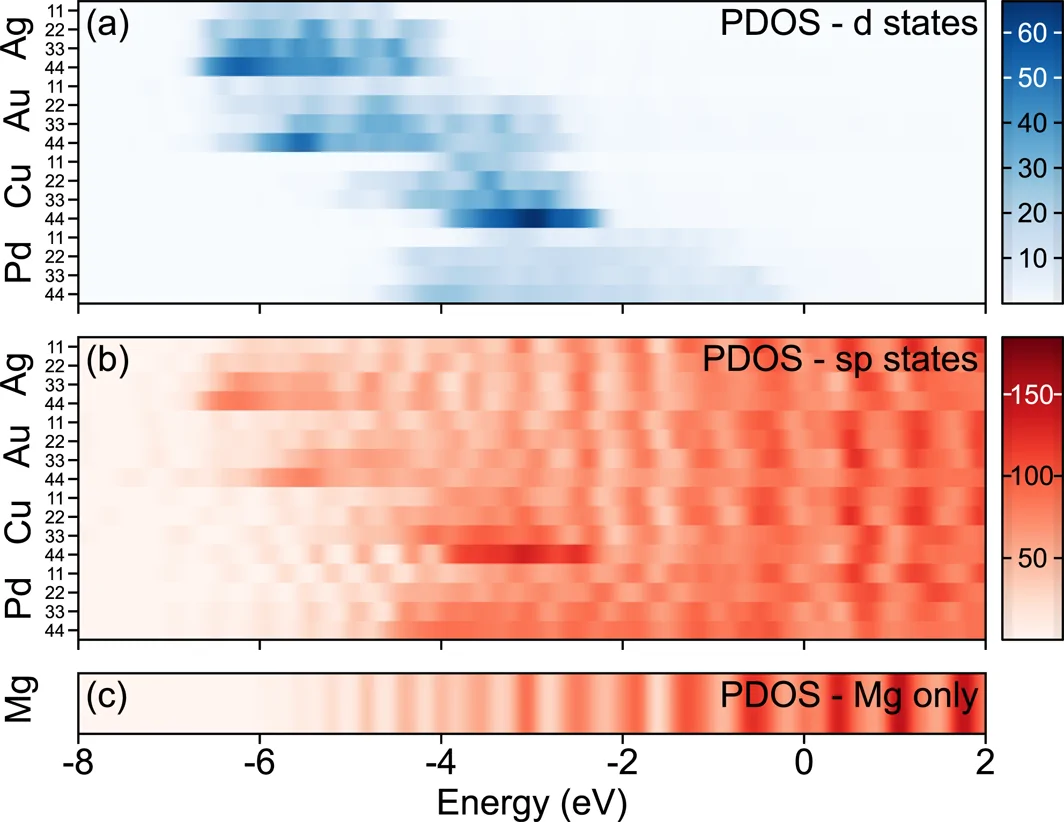
Partial density of states of doped $A_{x}{\text{Mg}}_{309\mathrm{-x}}$ nanoparticles decomposed into (a) the d‐state contributions and (b) the sp‐states as function of the dopant number and type. Note the distinct mixing of the d‐states into the sp‐states of the doped NPs. (c) PDOS of the undoped Mg_309_ NP. The color map is the same as in (b).

The width of this d‐band Janus NPs differs according to the dopants type. The narrowest band is obtained for Cu, which is known from its electronic configuration with narrow d‐band structure [56]. It is important to note that d‐band created by Pd dopants extends up to the Fermi level, which is caused by not fully occupied d‐orbitals in Pd. This property differentiates it from transition metals such as Ag, Au, and Cu having fully occupied d‐orbitals [57]. We observe fingerprints of the d‐PDOS in the PDOS of the sp‐state proving significant hybridization. Fingerprints of this increasing hybridization to the sp‐states with the number of dopant atoms are seen in the strongly increasing mixed transitions shown in Figure 2 and in the significant increase in their intensity with the number of dopant atoms. Also, the impact of broadening of the d‐states is visible in the red‐shift of the dopant→Mg transitions, as higher lying d‐electrons can be excited with increasingly lower energies to the unoccupied states in the host atoms. The stronger dopant→Mg mixing observed for Pd, whose d‐band extends to the Fermi level, correlates with its markedly larger dopant→Mg transition contributions compared to Ag, Au, and Cu.

### 3.2 Hot‐Carrier Generation

In the realm of HC, the interactions between electrons and the external excitation play a key role in determining HC energies. Different dopants can affect the strength of these interactions, impacting the efficiency of energy transfer and consequently affecting the amount of generated HC [28, 58]. In addition to the type of dopant, the spatial localization of dopant atoms, particularly at the host‐dopant interface, influences the generation of hot carriers within the nanoparticle. In Figure 5, we show the HC energy distributions as a function of the excitation pulse energy projected into dopants and host states in NPs containing 44 atoms of dopants (the yellow line in each plot denotes the respective PDOS of the particular part of the doped NP). Due to the secondary photoabsorption spectra induced by the dopant electronic states, the intensity of HC generation displays two distinct maxima. These maxima correspond directly to the resonance frequencies at 4.90 and 6.00 eV. The HC generation probability relates to the absorption intensity; therefore, most of the HC are generated for the excitation energies around 4.90 eV.

**FIGURE 5:**
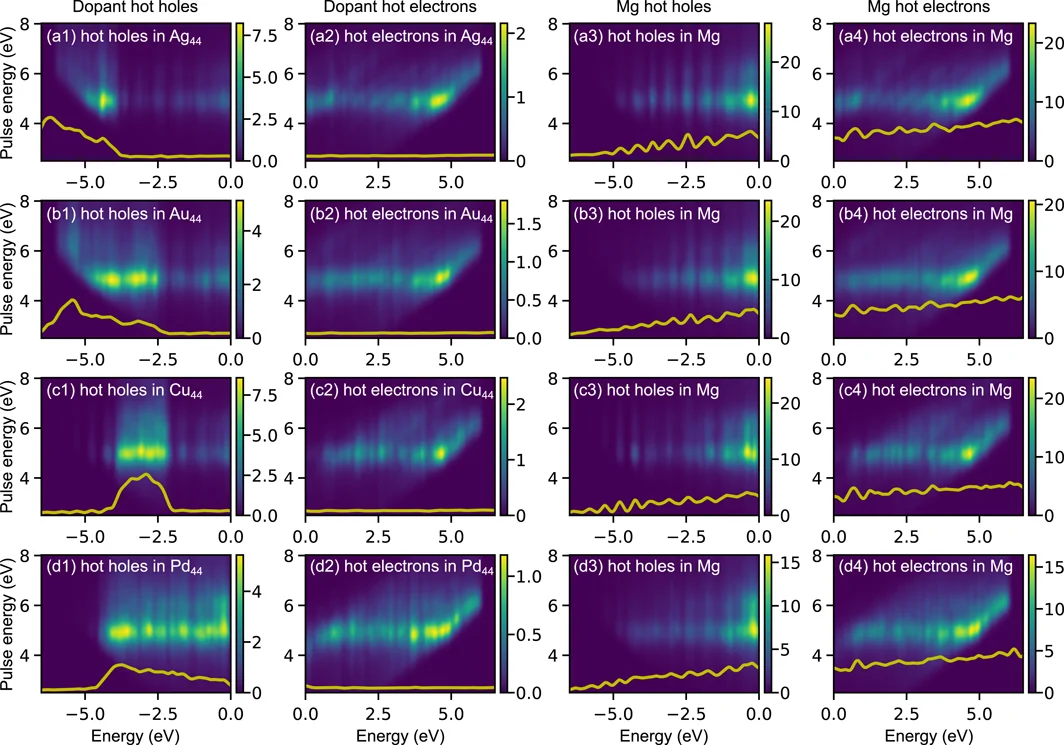
Hot carrier generation in doped Mg_309_ NPs with 44‐dopant atoms versus carrier energy (x‐axis, Fermi energy is at 0 eV) and excitation pulse frequency (y‐axis) in Janus heterostructures. In row (a) Ag_44_Mg_265_, in (b) Au_44_Mg_265_, in (c) Ag_44_Mg_265_, and in (d) Pd_44_Mg_265_ with columns (1,2) showing dopant hot holes and electrons, respectively, and columns (3,4) the same for the Mg remainder. The line plots in each panel denote the particular PDOS for the dopant or host Mg. The hot carriers in Mg are in every case characterized by a similar spectral distribution with the biggest signal around the LSPR frequency at 4.9 eV, whereas the hot holes and electrons, respectively, appear predominantly just below the Fermi energy and just below 5 eV. The dopants' largest contribution to excited carriers is in the form of hot holes whose energies are aligned to their respective d‐states. In contrast, the dopants' hot electrons mirror that of Mg, showing the strong mixing of the sp‐states of the doped system.

We can clearly see that hot holes' energy distribution changes with the dopant type in the Janus heterostructures. It can be explained by the differences in DOS in particular PDOS of d‐band energy states (indicated with yellow solid lines), as the majority of hot holes are localized on the dopant atoms particularly those at the interface with the Mg host (see subsequent discussion). In contrast, the hot electrons' energy distribution is similar in both host and dopant atoms, indicating that the hot electron states remain delocalized over the whole NP. There are small differences in hot electron energy distribution between NPs with distinct dopants, what is caused by alignment of occupied and unoccupied states within the structure. We observe slight shifts and width changes of the hot electron generation probability maxima that correlate with the position and width of d‐bands. In particular, the maxima in Mg NP doped with Cu is the narrowest, and in Mg NP doped with Pd is the widest, reflecting the narrower Cu d‐band and broader Pd d‐band, respectively (Figure 4). Similarly, deep d‐states present in Ag and Au result in an increased probability of generation of hot electrons with energy slightly above the Fermi level. In the case of random heterostructures, the correlation between d‐states and hot hole energy distribution is more pronounced due to their narrow d‐states PDOS, see Supporting Information S1: Figure S3. In these random cases, the hot hole energy is spectrally fixed by the position of the d‐states and offers less tunability. Such relations indicate the presence of mixed electron transitions between dopant and host within the NPs and the relevant role of the spatial distribution of the dopant atoms in the heterostructure.

Notably, an analysis of the overlap of hot holes with the PDOS reveals distinctive features. The intersection of the hot hole distribution with PDOS of dopants d‐electrons shows that not all d‐electrons undergo the transfer process (Figure 5). This selective participation of d‐states is directly reflected in the specific optical transitions of elements shown in Figure 2 where only certain energy ranges exhibit significant contributions from dopants, such as those driven by the efficient LSPR excitation. This can be explained by the fact that the energy levels of the d‐electrons in the dopant atoms may not align with the free energy states available in the NP electronic structure, preventing efficient transfer. As seen in Figure 5, for Ag and Au, we found an overlap of hot holes with a limited portion of the PDOS electrons, where only a fraction of dopant d‐electrons is directly involved in electron transfer. In the case of these dopants, the electrons are located at lower energy levels and therefore face difficulties in finding higher free energy states when the NP is driven around the LSPR energy (ca. 4.9 eV). However, these states become activated following an increase of the excitation pulse energy above the LSPR, as we observe a growth in d‐electron hole generation and thus transfer intensity. Despite this increase, the hot hole generation efficiency is markedly lower than at the LSPR peak.

This demonstrates how disparities in energy levels between the dopant and host material influence electron transfer and subsequently impact the distribution of HC. In addition to energy alignment, the spatial position of dopant atoms, whether at the interface with Mg or within the dopant domain, plays a role in determining the efficiency of hot carrier generation. The results highlight the importance of considerations linked to the energy level and atomic arrangement for understanding the distribution of HC within the atoms of doped NPs. Additionally, an analysis of the distribution of hot electrons in the dopant compared to that of Mg atoms indicates the presence of shared/hybridized bright regions between the dopant and Mg (Figure 5). The presence of these bright regions suggests the hybridization of their respective sp states, signifying a shared spatial position for hot electrons in the Janus heterostructures (c.f. Columns 2 and 4 in Figure 5). Notably, this hybridization is most pronounced for dopant atoms located at the hot‐dopant interface where orbital overlap is maximized. Interestingly, for the random heterostructures, hot electron spectra in Supporting Information S1: Figure S3 show a strong increase for Cu and Pd at low energies both in the dopant and host Mg atoms. This is a direct consequence of exciting electrons from the narrow d‐band of the dopant rather than a broad one in the Janus heterostructure case. These observations provide compelling evidence for the interaction and hybridization of electronic states between the dopant and Mg, highlighting the complex dynamics of their hot electron behavior [59]. Doping‐induced changes in Mg NP highlight the impact of dopant elements on the dynamics of HC generation where the discrete delocalized electronic states of the dopant atoms play a crucial role in shaping the local HC landscape. This deduction suggests a new approach for tailoring HC dynamics through strategic doping and an interplay with the LSPR of the host NP.

Although we have discussed above the case for the maximally doped NP, we also consider the influence of the number of dopant atoms on HC energy distributions. These data are plotted for the hot holes in Figure 6 (variations in the hot electron distribution in the dopants and for both hot holes and electrons in the host Mg are much smaller and omitted). From these data, it is clear that the degree of doping, which introduces a variable amount of d‐electron states not only adds new plasmon decay pathways but also changes the energy distribution of generated HC and simultaneously increases the number of dopant atoms located at the interface between the dopant domain and the host Mg, thereby enhancing the spatial contribution of interfacial sites to the observed trends. The Mg NP LSPR is the dominant spectral feature that drives absorption and hot carrier generation. However, a larger degree of doping increases the mixing of electronic states at the dopant‐Mg interface and the hot carrier especially the hot hole, spectra become less discrete and typically broaden spectrally. Interestingly, for Ag and Au, whose d‐electron PDOS extends lower in energy, we observe increased generation of hot holes when driven with a pulse above the main LSPR peak (≳6 eV). This is observed especially for smaller doping, while for larger doping the hot hole spectra are dominated by the LSPR of the Janus heterostructures. We compare these results with those of the random heterostructure with 44 dopant atoms, which is plotted in Figure 6e, whose d‐band PDOS is sharper and more intense than for the Janus counterparts. It results in highly localized hot holes concentrated within a narrow energy range, which limits spectral tunability and is expected to be unfavorable for applications such as solar energy driven photocatalysis.

**FIGURE 6:**
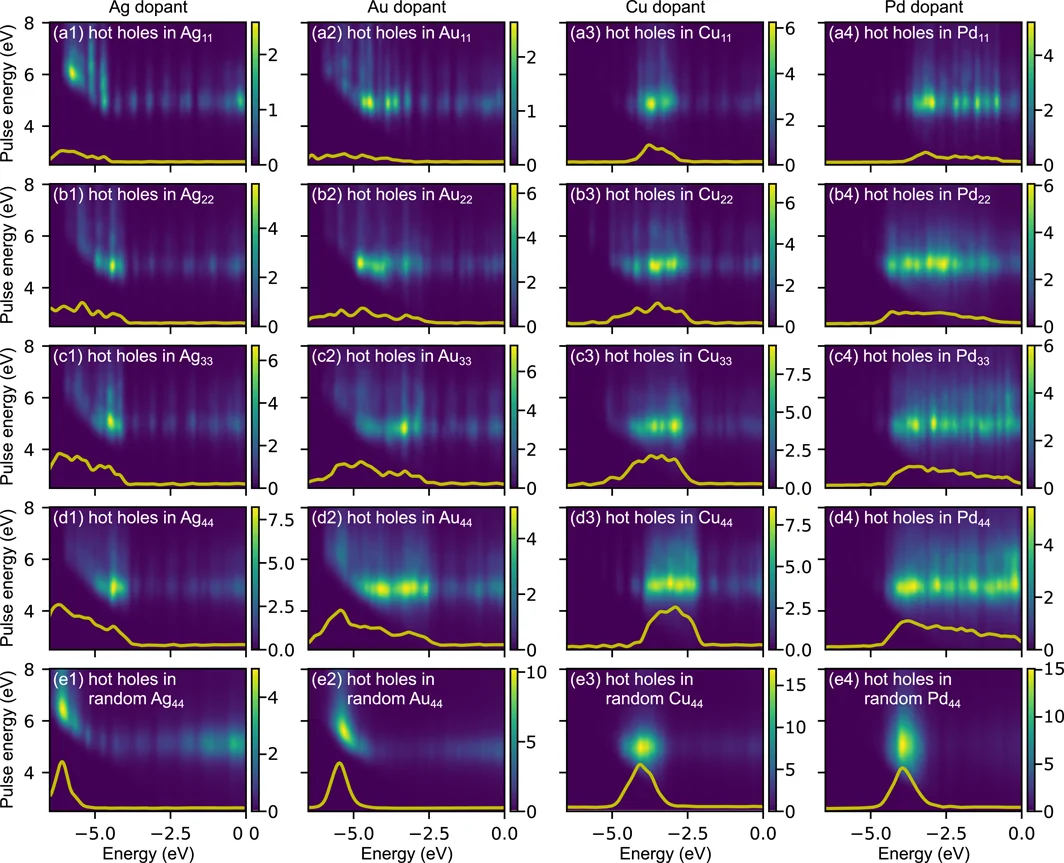
Changes of spectra of hot holes generated in the dopant atoms versus carrier energy (x‐axis, Fermi energy is at 0 eV) and excitation pulse frequency (y‐axis); the line plots in each panel denote the PDOS for the dopant. (a–d) Janus heterostructures: in row (a) for X_11_Mg_298_, in (b) X22Mg287, in (c) X_33_Mg_276_, and in (d) X_44_Mg_265_ with columns showing different dopant atoms. (e) Random heterostructure NPs with 44 dopant atoms show significantly localized d‐states than the Janus heterostructures.

In Figure 7, we plot the spectral dependence of the total amount of hot carriers (electrons with the dashed line; holes with the dotted line) generated in the two constituent subparts of the doped Janus heterostructure NPs as function of the excitation pulse energy. The result shows that a transition from Ag, having low d‐election states, to Pd, having high d‐electron states, results in an increase of the HC separation. This separation, which is plotted independently for the host Mg part (Figure 7a,c,d,g) and dopants (Figure 7b,g,f,h), is highlighted by the colored areas, which mark differences between amounts of hot electrons and holes in the two subparts. By examining changes in HC amounts generated in response to the external excitation, we observe a correlation with the shape of the photoabsorption spectra especially the LSPR. As a result, the evolution of the external excitation of the pulsed energy induces distinct transitions to the unoccupied states [24]. The association between dopant type and concentration affects the energy levels available to HC, thereby influencing their distribution within the Janus NP. In the case of random heterostructures, the generation and separation of hot carriers is similar (to that in Janus NPs) in magnitude for Cu and Pd while is smaller for Ag and Au, cf. Supporting Information S1: Figure S4. The Cu and Pd spectra (Fig. S4e‐h) follow closely the LSPR and do not exhibit a shoulder at larger energies (cf. Figure 7e–h). In contrast, the spectra for Ag and Au with their deeper d‐states exhibit hot carrier generation both at the LSPR peak and at its high‐energy tail.

**FIGURE 7:**
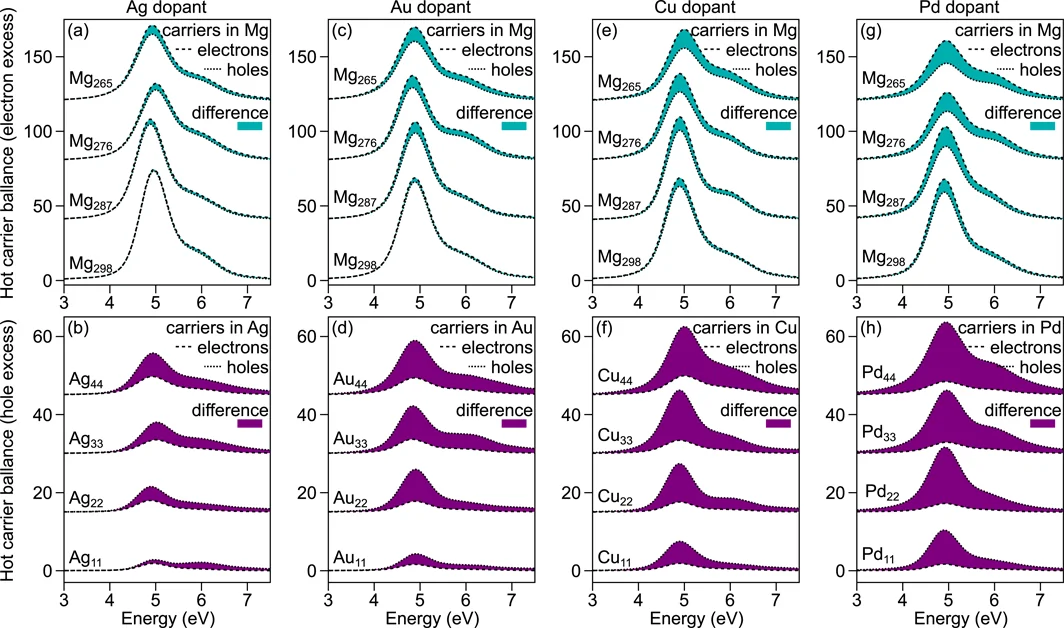
Total number of hot carriers (dashed—electrons; dotted—holes) for Janus heterostructure nanoparticles with different dopant composition (type and number) versus excitation pulse energy from 3 to 7.5 eV. The colored area marks the difference in hot carriers with (a, c, e, g) cyan denoting excess hot electrons and (b, d, f, h) magenta an excess of hot holes. The amount of excess hot holes in the dopant is equal to the excess hot electrons in the host Mg. (a, b) Ag dopant, (c, d) Au dopant, (e, f) Cu dopant, (g, h) Pd dopant.

We now turn to evaluate the impact of the spatial arrangement of dopant atoms on HC generation, which are expected to determine the efficiency and localization of hot hole generation. We illustrate these dependencies in Figure 8 by plotting the hot hole excess normalized per dopant atom and decomposed by local atomic environment for random (top row) and Janus heterostructures (bottom row) for 44 dopant atoms. Dopant atoms are classified into three groups: *surface dopant* atoms surrounded only by other dopant atoms, *surface interface dopant* atoms at the surface with at least one Mg neighbor, and *bulk dopant* atoms. In the random heterostructure NPs, the hot hole excess in the interface dopant atoms is always larger than in the bulk, but the differences are small. The slightly increased hot hole generation at the surface reflects the enhancement of near electromagnetic field (see Figure 9f), which in the random heterostructures is relatively small and uniform. We note here that during relaxation surface dopant atoms in the random heterostructures have a tendency to shift slightly towards the interior of the NP.

**FIGURE 8:**
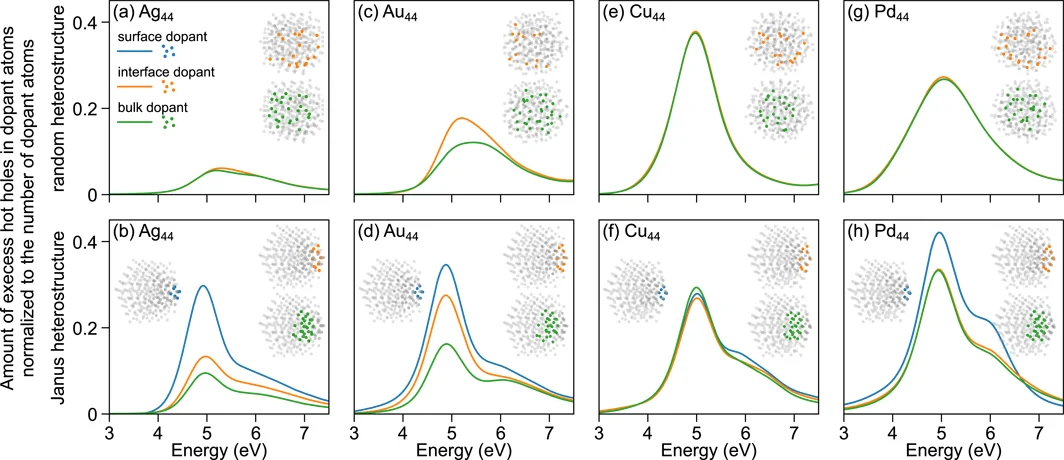
Hot hole excess in dopant atoms in (a, c, e, g) randomly alloyed NPs and (b, d, f, h) Janus type NPs with 44 dopant atoms per dopant atom. (a, b) Ag_44_ dopant, (c, d) Au_44_ dopant, (e, f) Cu_44_ dopant, (g, h) Pd_44_ dopant. Dopant atoms are divided into 3 groups depending on their neighborhood: surface dopant atoms surrounded by only dopant atoms (color blue), interface surface dopant atoms surrounded by at least one Mg atom (color orange), and bulk dopant atoms (color blue). The insets show the position of dopant atoms of particular group in the NP.

**FIGURE 9:**
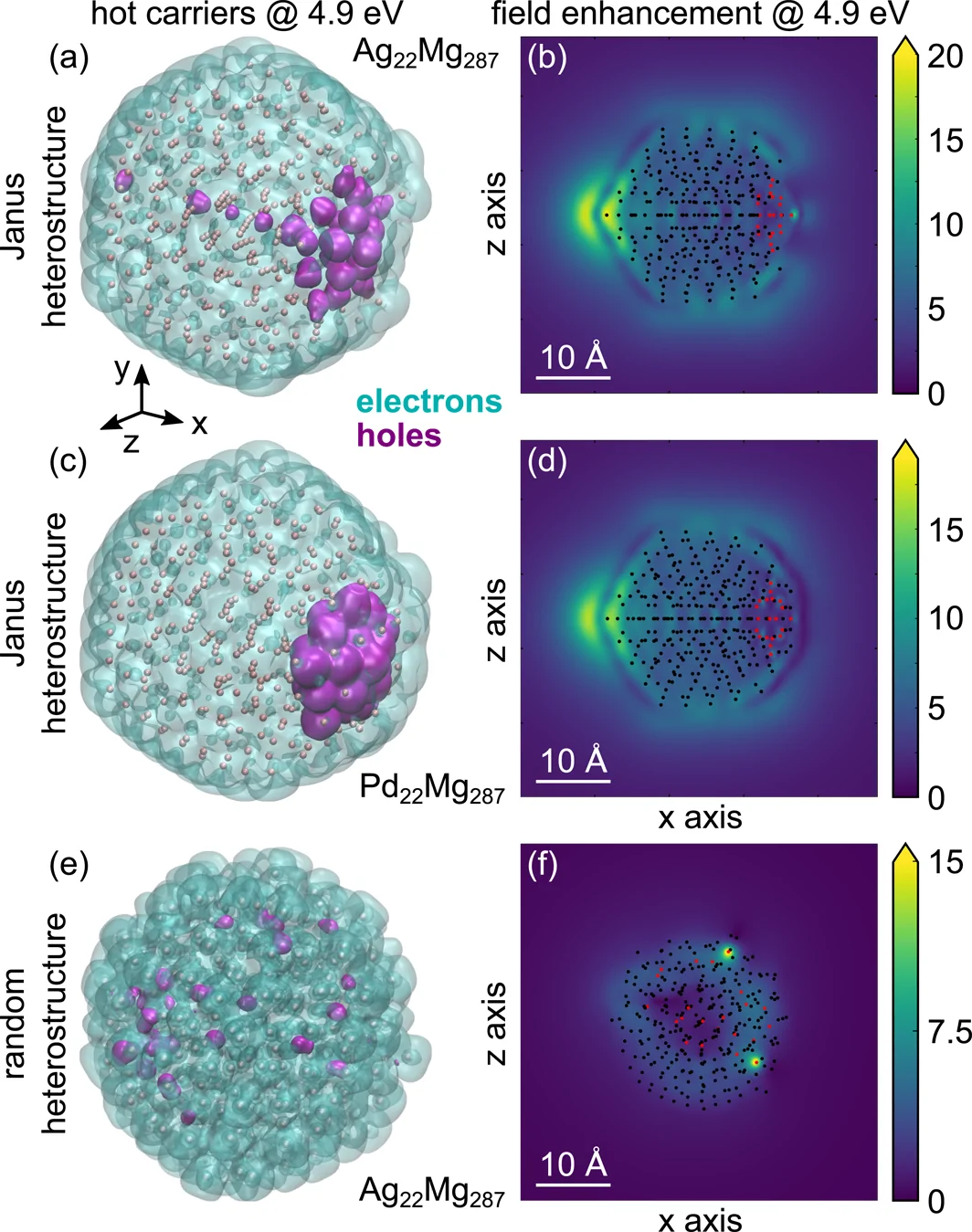
Spatial distribution of (a, c, e) hot carriers and (b, d, f) induced electric fields at resonance 4.9 eV for selected doped clusters (a, b) Janus NP Ag_22_Mg_287_, (c, d) Janus NP Pd_22_Mg_287_, and (e, f) random NP Ag_22_Mg_287_. The hot holes (magenta) are localized to the dopant atoms, while the hot electrons (cyan) are distributed uniformly throughout the host Mg. The induced electric field is enhanced relative to the incident one along the polarization direction (*x*‐axis), although the dopant atoms (marked in red) decrease its intensity converting it to excited holes. The Mg atoms are marked in black.

In the Janus heterostructures, the hot hole generation efficiency is the largest for the surface dopant atoms (fully surrounded by other dopant atoms) followed by the surface interface dopant atoms with the bulk dopants having the smallest efficiency. The differences between the three categories can be large, especially in Ag and Au dopants, highlighting the distinct local environment created by a spatially segregated dopant domain. Hot holes are preferentially distributed at surface dopant atoms surrounded by other dopants, which argues that phase separated Janus heterostructures result in localized and intense hot hole distributions. For Ag and Au, the Janus configuration systematically yields a larger hot hole excess per dopant atom than the random heterostructures, demonstrating that spatially segregated dopant domains are more efficient hot hole generation sites. The same is true also for Pd, although the advantage of the ordered Pd Janus‐NP over the random doping is not as large as for Ag and Au. Cu, on the other hand, represents an exception where the random heterostructure yields a slightly higher excess than the Janus‐type NP, suggesting that the local atomic environment has a distinct effect on Cu hot hole generation.

This contrasting behavior can be explained by the intrinsically narrower d‐band of Cu compared to Ag, Au, and Pd [56]. In the Janus structure, aggregation of dopants facilitates the hybridization between them resulting in a broader and more delocalized d‐band that interacts with Mg sp‐states and facilitates interband transitions. For Cu, the energy range of the d‐band matches well the LSPR of the Mg, ensuring efficient driving by the enhanced electric field of the NP, whereas for the other three dopants, its larger width also ensures adequate overlap with the plasmon. In contrast, in the random heterostructures the relative isolation of the dopant atoms results in narrow d‐bands (quasi‐isolated d‐states). In Ag and Au, it is detuned both from the sp‐states resulting in limited broadening and hybridization. It is also detuned from the LSPR and thus is weakly enhanced. For Pd and Cu in the random heterostructure, the d‐states are higher in energy and thus hybridize better with the sp‐states. More importantly, their localized d‐state character provides a favorable energetic match with LSPR excitation energies at 4.9 eV (see Figures 1d–g and 6), promoting interband transitions and hot hole generation. For Pd and especially Cu with slightly broader d‐states, this leads to slightly improved hot hole generation in the random distribution, indicating that the electronic interactions of spatially isolated dopants with the host are more favorable than those with clustered dopants. In Pd matching of the d‐states’ energy to the LSPR is not as good as for Cu, thus, it performs slightly worse.

Hence, controlling the spatial arrangement of dopant atoms, beyond element choice and concentration, offers a practical handle to tailor hot hole generation in bimetallic NPs. Furthermore, in the Janus NPs, confining dopant atoms to a small, spatially localized cluster modifies the d‐states compared to the random configuration in which the d‐states are spectrally confined, cf. Supporting Information S1: Figure S2. This smeared d‐state distribution in Janus heterostructures promotes interband transitions, a feature directly relevant for photocatalysis.

Figure 9 illustrates the variation of HC spatial distribution in heterostructures of various types and the corresponding modifications of the induced electric field enhancement. Placement of the dopant atoms in small clusters, as shown in Figure 9a,c, creates strongly confined distributions of the hot holes (magenta) in the dopants. The d orbitals incorporated into the NP structure create additional energy levels that attract and confine HC leading to a stronger localization at the dopant site compared to the rest of the NP [60]. This process is very efficiently driven by excitation of the localized plasmon, which enhances absorption and whose fingerprint can be found in the enhanced field in Figure 9b,d. The dopant atoms, which are located in the right part of the particles (red dots), are driven by the LSPR and convert the intense electric field to excited hot carriers, which are then spatially separated in the Janus heterostructures. Both the HC and electric field enhancement are significantly different for a random heterostructure, cf. Figure 9e,f. The random distribution of dopants produces a weaker electric field enhancement leading to smaller HC generation. Simultaneously, the hot holes, being confined to the dopant atoms, are spread out over the surface and volume of the randomly doped NP, making this configuration less suitable for efficient hot carrier harvesting and/or plasmon‐enhanced photocatalysis.

These observations highlight that both the spatial arrangement of dopant atoms and their electronic structure collectively determine the hot carrier distribution. Although the spatial arrangement of HC remains relatively consistent across dopants, the energy characteristics and localization patterns of hot holes are significantly influenced by the choice of dopant material and its position within the heterostructure. This understanding is crucial for tailoring NP properties for specific applications in fields such as catalysis or optoelectronics where controlling the location of hot carriers at reactive sites is essential.

## 4 Conclusions

We showed that hot carrier generation in bimetallic nanoparticles is governed by interfacial electronic states, leading to spatially localized hot electrons and holes. Doping Mg with d‐electron‐rich elements induces a d‐band that acts as an electron reservoir, promoting hot hole formation at the Mg‐dopant interface and surface, explaining the preferential localization of hot holes at the interface rather than within the dopant. Such spatial selectivity is critical for photocatalysis, where hot holes drive oxidation and hot electrons enable reduction together supporting full redox cycles for solar energy conversion. The energy distribution of hot electrons reflects the width of d‐band giving the possibility of tailoring the energy range of generated hot electrons. Moreover, the projected density of d‐states changes with the number of dopant atoms and their spatial distribution. Specifically, the d‐band, whose electronic properties are crucial to efficient generation of tailored hot holes, can be tuned by changing the way dopant atoms are distributed in the host nanoparticle. For randomly placed dopant atoms, the d‐band remains relatively narrow, but its energy width and position can be tuned by spatially arranging dopants into few‐atom clusters. Depending on the requirements to match the energy of hot carriers to a particular reaction, the same number of dopant atoms can be arranged into a single large or a few smaller dopant clusters to ensure efficient HC generation. Such engineering will yield optimum use of the dopant atoms to generate spectrally tailored and spatially separated hot carriers. We observe also that the increase of mixed dopant–host and host‐dopant transitions drops down with increasing number of dopant atoms, due to the reduced fraction of interfacial atoms, indicating that hot‐carrier generation is primarily controlled by the spatial distribution of interfacial dopants rather than their total amount. Interestingly, regardless of the dopant type second absorption resonance appears in higher energies around 6 eV. This insensitivity of absorption frequency to dopant type indicates that we observe an enhancement of a host NP resonance rather than a new plasmon excitation created in dopant domain. This lack of a significant change in the position of LSPR means that the optimal energy of excitation pulse to maximize the probability of HC generation remains the same. Finally, we showed a direct link between dopant spatial arrangement and hot‐carrier localization, showing that interfacial atomic distribution governs both electronic structure and excitation pathways enabling controlled hot‐carrier generation.

## Author Contributions

**Rania Zaier:** writing – original draft, visualization, investigation, writing – review and editing, formal analysis, methodology. **Katarzyna Kluczyk‐Korch:** writing – review and editing, validation, investigation, writing – original draft, formal analysis, methodology, visualization. **Tomasz J. Antosiewicz:** conceptualization, investigation, funding acquisition, writing – review and editing, visualization, supervision, resources, validation, project administration, data curation, formal analysis, methodology.

## Supporting information


Supporting Information S1

## Data Availability

The data that support the findings of this study are available from the corresponding author upon reasonable request.
