# Supplementary material for: Interfacial Role of Dopant d‐States on Hot Carrier Generation in Bimetallic Nanoparticles
Source: Nanophotonics. 2026 Jul 1;15(13):e70184. doi: 10.1002/nap2.70184 (PMC13642517; doi:10.1002/nap2.70184)
Supplement: Supplementary file 1 [file NAP2-15-e70184-s001.pdf]

Supporting Information for

Interfacial role of dopant d-states on hot carrier generation in bimetallic nanoparticles

Rania Zaier[1,†], Katarzyna Kluczyk-Korch[1,*], and Tomasz J. Antosiewicz[1,2,‡]

[1] *Faculty of Physics, University of Warsaw, Pasteura 5, 02-093, Warsaw, Poland*
[2] *Department of Physics, Chalmers University of Technology, 412 96, Göteborg, Sweden*
[†]email: rania.zaier02@gmail.com
[*]email: katarzyna.kluczyk@fuw.edu.pl
[‡]email: tomasz.antosiewicz@fuw.edu.pl

Contents

Supplementary Note 1: Spectra of nanoparticles with randomly distributed dopant atoms **2**

Supplementary Note 2: Partial density of states of random heterostructures **2**

Supplementary Note 3: Hot carrier energy distributions in random **3**

Supplementary Note 1: Spectra of nanoparticles with randomly distributed dopant atoms.

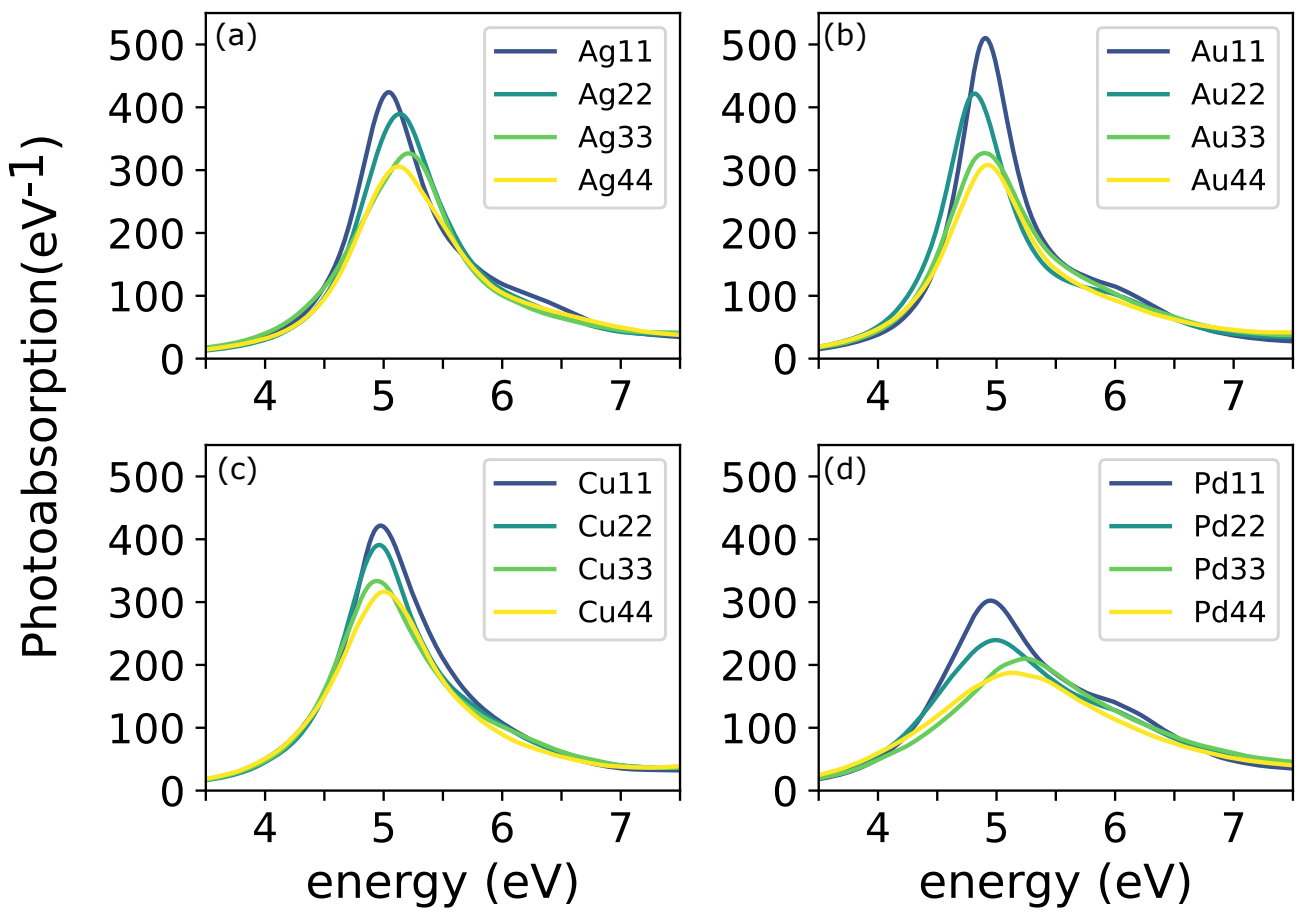

Figure S1: Photoabsorption spectra of Mg nanoparticles with random distribution of dopant atoms. (a) Photoabsorption spectrum of $Mg_{309-x}Ag_x$ nanoparticles. (a) Photoabsorption spectrum of $Mg_{309-x}Au_x$ nanoparticles. (c) Photoabsorption spectrum of $Mg_{309-x}Cu_x$ nanoparticles. (d) Photoabsorption spectrum of $Mg_{309-x}Pd_x$ nanoparticles.

Supplementary Note 2: Partial density of states of random heterostructures.

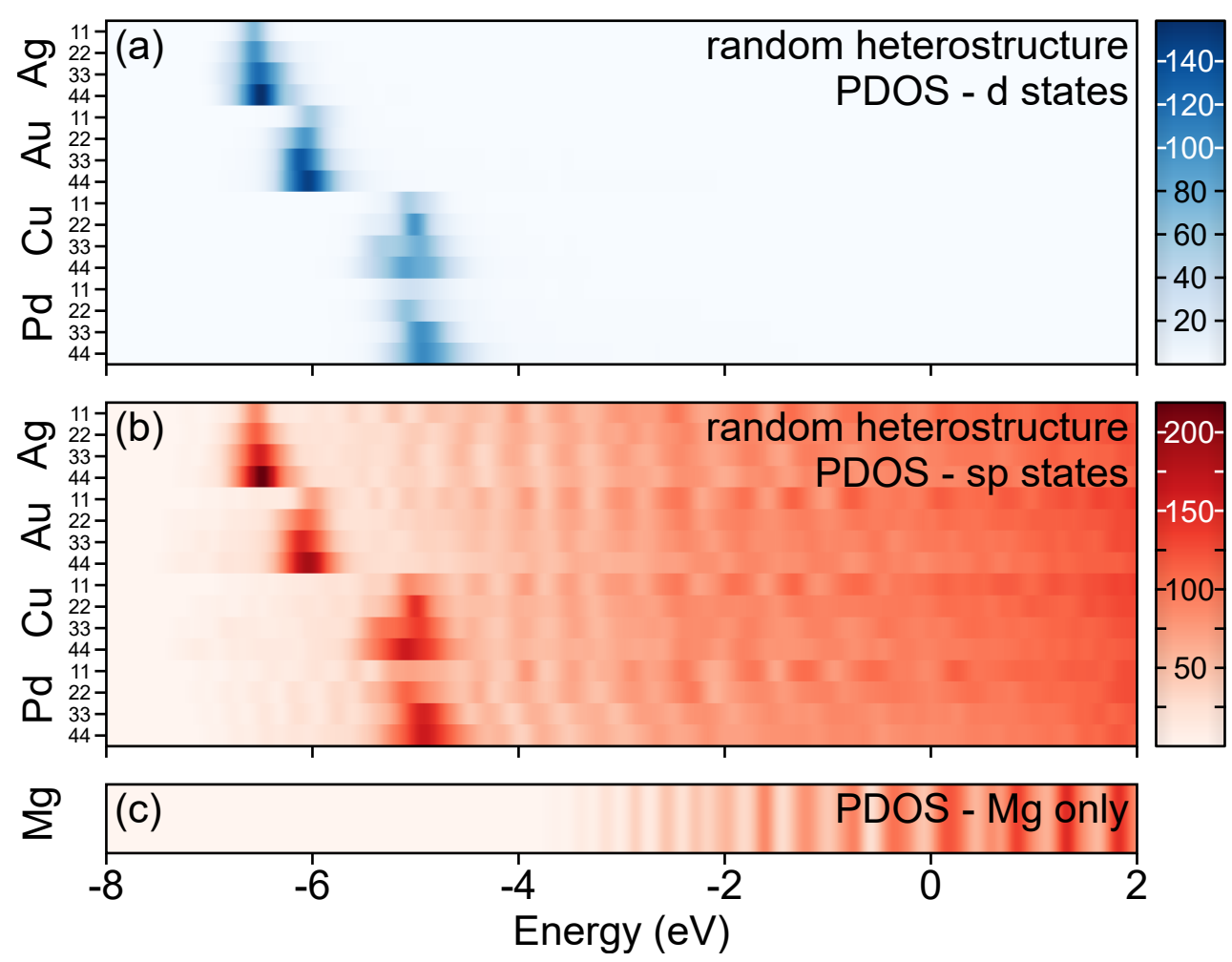

Figure S2: Partial density of states of random heterostructures alloyed nanoparticles decomposed into (a) the *d*-state contributions and (b) the *sp*-states as function of the dopant number and type. Note the distinct mixing of the *d*-states into the *sp*-states of the doped NPs.

Supplementary Note 3: Hot carrier energy distributions in random .

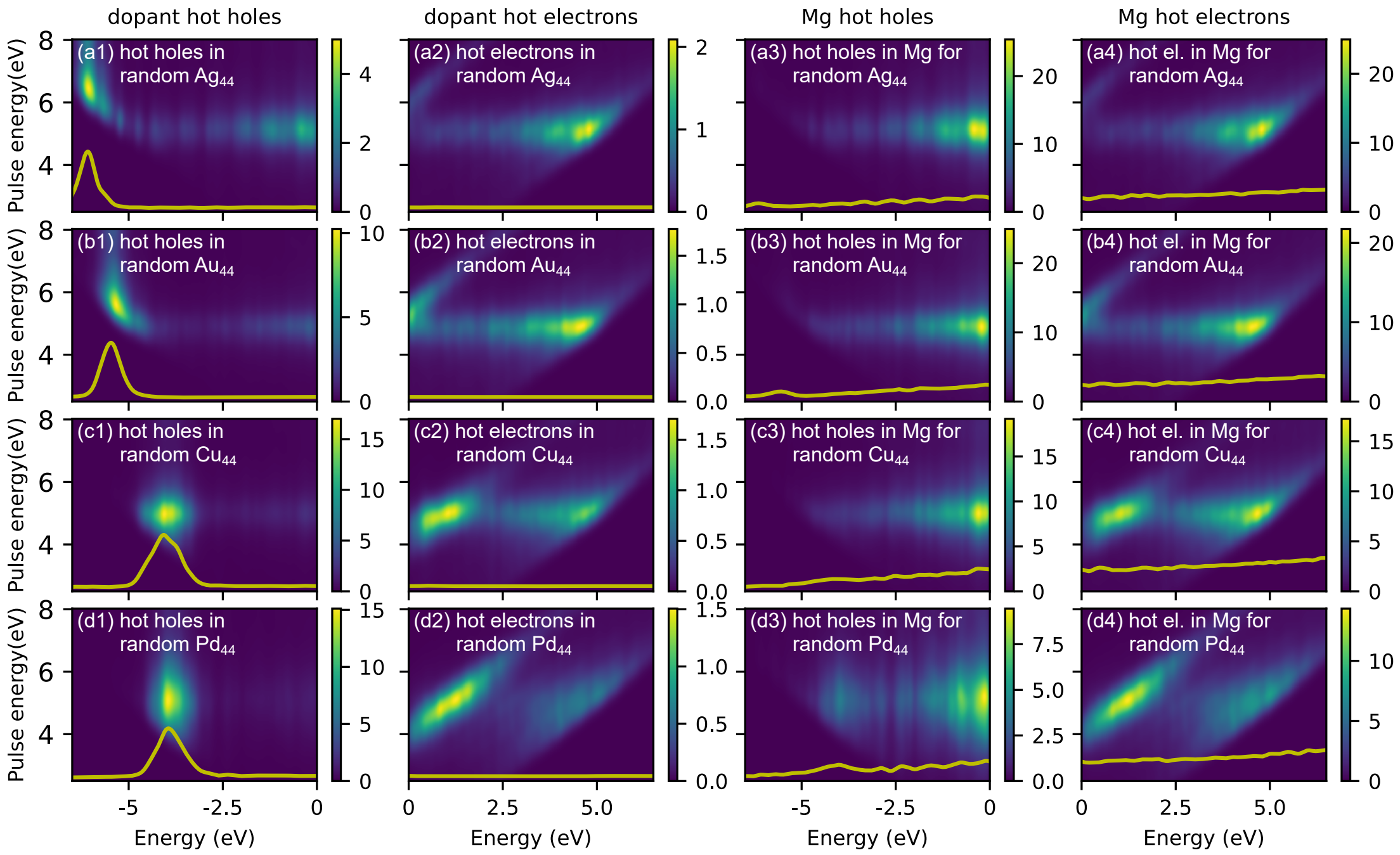

Figure S3: Hot carrier generation in random heterostructures based on Mg_{309} NP with 44-dopant atoms versus carrier energy (x-axis, Fermi energy is at 0 eV) and excitation pulse frequency (y-axis). In row (a) $Ag_{44}Mg_{265}$, in (b) $Au_{44}Mg_{265}$, in (c) $Cu_{44}Mg_{265}$, and in (d) $Pd_{44}Mg_{265}$ with columns (1,2) showing dopant hot holes and electrons, respectively, and columns (3,4) the same for the Mg remainder. The line plots in each panel denote the particular PDOS for the dopant or host Mg. The hot carriers in Mg are in characterized by a similar spectral distribution with the biggest signal around the LSPR frequency at 5.1 eV. The hot holes energy predominantly lays slightly below Fermi energy for Ag, Au and Cu. In case of Pd, we observe additional peak in hot holes energy distribution around 3.8 eV. This is reflected in hot electron energy distribution with one peak just below 5 eV (similarly as in Janus heterostructures) and second gradually appearing in Ag and Au just above Fermi level, through Cu with maximum just below 1 eV to Pd with maximum slightly above 1 eV. The second peak depicts transitions originating from deep narrow *d*-states present in randomly alloyed structures. The dopants' largest contribution to excited carriers is in the form of hot holes whose energies are aligned to their respective *d*-states. In contrast, the dopants' hot electrons mirror that of Mg, showing the strong mixing of the sp-states of the doped system.

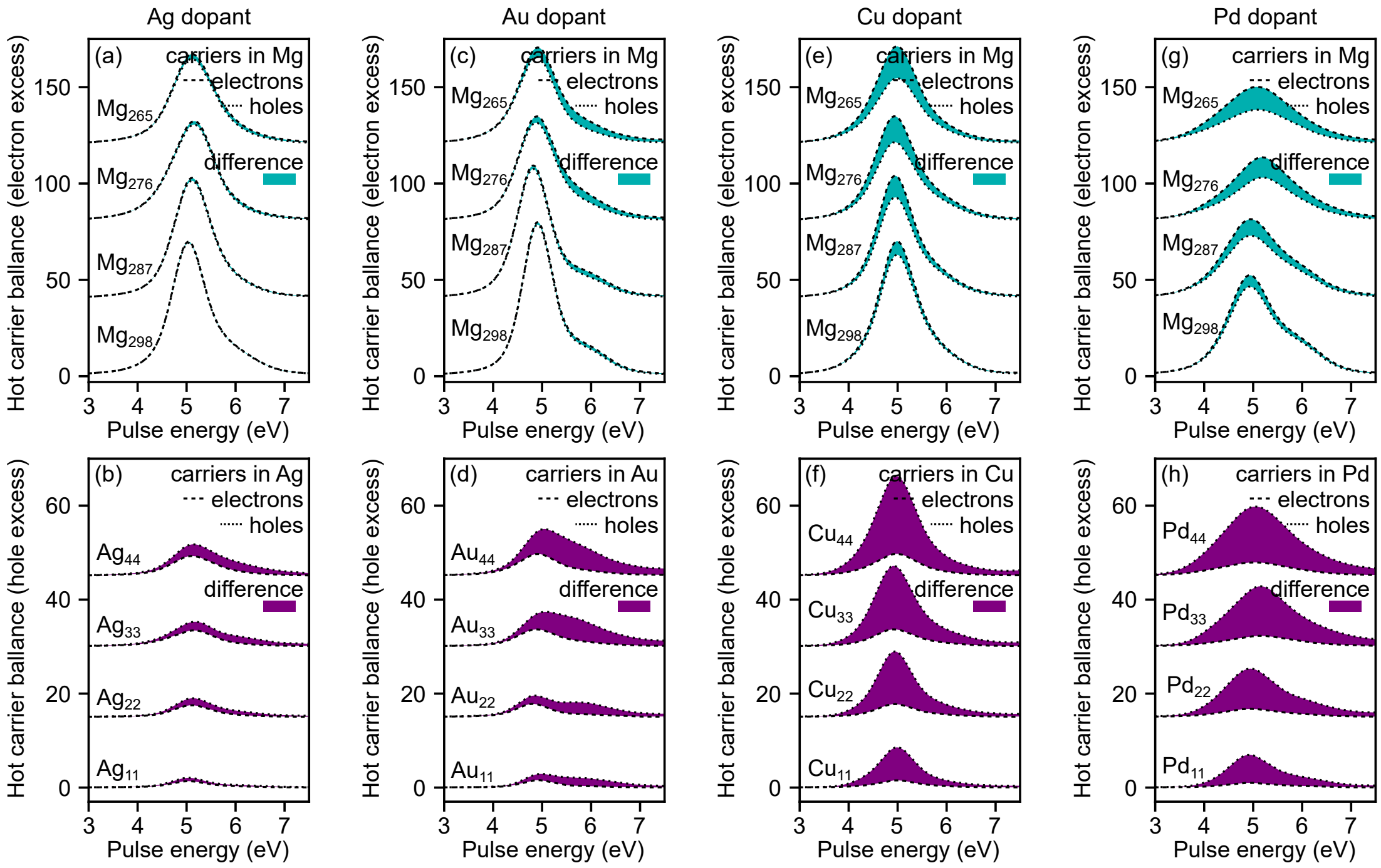

Figure S4: Hot carrier generation in random heterostructures. Total number of hot carriers (dashed – electrons; dotted – holes) for NPs with different dopant composition (type and number) versus excitation pulse energy from 3 to 7.5 eV. The coloured area marks the difference in hot carriers with cyan denoting excess hot electrons and magenta an excess of hot hole. The amount of excess hot holes in the dopant is equal to the excess hot electrons in the host Mg.
